# Biomechanical Comparison of Multilevel Lumbar Instrumented Fusions in Adult Spinal Deformity According to the Upper and Lower Fusion Levels: A Finite Element Analysis

**DOI:** 10.1155/2022/2534350

**Published:** 2022-11-30

**Authors:** Dong-Min Son, Soo-Bin Lee, Sung-Jae Lee, Tae-Hyun Park, Ji Eun Jang, Seung Jo Jeong, Young-Mi Kang, Byung Ho Lee

**Affiliations:** ^1^Department of Biomedical Engineering, College of Biomedical Science & Engineering, Inje University, 197 Inje-ro, Gimhae-si Gyeongsangnam-do 50834, Republic of Korea; ^2^Department of Orthopedic Surgery, Catholic Kwandong University International St. Mary's Hospital, 25, Simgok-ro 100beon-gil, Seo-gu, Incheon 22711, Republic of Korea; ^3^GS Medical Co., Ltd., 90 Osongsaengmyeong 4-ro, Osong-eup, Heungdeok-gu, Cheongju-si, Chungcheongbuk-do 28161, Republic of Korea; ^4^Department of Orthopedic Surgery, Yonsei University College of Medicine, 50 Yonsei-ro, Seodaemun-gu, Seoul 03722, Republic of Korea

## Abstract

Multilevel lumbar fusion with posterior pedicle screw fixation is a widely performed surgical procedure for the management of adult spinal deformity. However, there has not been a comprehensive biomechanical study on the different types of fusion levels in terms of stability and possible complications. We aimed to investigate the biomechanical properties of multilevel lumbar fusion according to different types of upper and lower fusion levels. Six different types of fusions were performed using three-dimensional finite element models. Type A and B referred to the group of which upper fusion level was L1 and T10, respectively. Subtype 1, 2, and 3 referred to the group of which lower fusion level was L5, S1, and ilium, respectively (A1, L1-L5; A2, L1-S1; A3, L1-ilium; B1, T10-L5; B2, T10-S1; B3, T10-ilium). Flexion, extension, axial rotation, and lateral bending moments were applied, and the risk of screw loosening and failure and adjacent segment degeneration (ASD) was analyzed. Stress at the bone-screw interface of type B3 was lowest in overall motions. The risk of screw failure showed increasing pattern as the upper and lower levels extended in all motions. Proximal range of motion (ROM) increased as the lower fusion level changed from L5 to S1 and the ilium. For axial rotation, type B3 showed higher proximal ROM (16.2°) than type A3 (11.8°). In multilevel lumbar fusion surgery for adult spinal deformity, adding iliac screws and increasing the fusion level to T10-ilium may lower the risk of screw loosening. In terms of screw failure and proximal ASD, however, T10-ilium fusion has a higher potential risk compared with other fusion types. These results will contribute for surgeons to provide adequate patient education regarding screw failure and proximal ASD, when performing multilevel lumbar fusion.

## 1. Introduction

Multilevel lumbar fusion with posterior pedicle screw fixation is a widely performed surgical procedure for the management of adult spinal deformity [1]. As global life expectancy is continuously increasing, and due to the development of medical care, the incidence and prevalence of adult spinal deformity are on the rise [2–4]. Previously, conservative management was recommended owing to higher morbidity associated with surgery [2]; however, surgical intervention with multilevel lumbar fusion has become the mainstay of adult spinal deformity treatment recently [4]. Numerous literatures have reported the superiority of multilevel lumbar fusion for the treatment of various types of adult spinal deformity [1, 5–9].

Multilevel lumbar fusion surgery has the advantage of spinal alignment correction; however, complications such as screw breakage and loosening or adjacent segment degeneration may occur. To avoid these complications associated with lumbar fusion surgery, artificial disc replacement (ADR) was developed for degenerative lumbar spinal diseases which do not require alignment correction. In a study using finite element analysis, single level ADR showed satisfactory results; however, multilevel ADR showed hypermobility which may lead to alter natural movement of spine and implant failure [10]. In clinical practice, whether ADR is superior to fusion is still controversial. To date, there is no other alternative than multilevel fusion surgery for adult spinal deformities requiring spinal alignment correction.

However, to our knowledge, there has not been a comprehensive biomechanical study on the different types of fusion levels in terms of stability and possible complications. Most previous studies were confined to complications about L5-S1 segment or proximal adjacent segment [11–14]. Furthermore, most studies on multilevel lumbar fusion were cadaveric experiments or clinical observational studies with a small sample size [13, 15, 16]. In this study, we aimed to investigate the biomechanical properties of multilevel lumbar fusion according to the different types of upper and lower fusion levels.

Using finite element analysis, we therefore asked: (1) How different is the risk of screw loosening between the fusion types? (2) How different is the risk of screw failure between the fusion types? (3) How different is the risk of adjacent segment degeneration (ASD) between the fusion types?

## 2. Materials and Methods

Ethical approval for this study was obtained from the Institutional Review Board (IRB) of the corresponding author's hospital (Yonsei University IRB and Ethics Committee: 4-2020-0060). All methods were performed in accordance with the Declaration of Helsinki and Yonsei University institutional guidelines. Informed consent was obtained from the subject.

### 2.1. Finite Element Analysis of an Intact Thoracolumbar Spine and Pelvis Model

**Figure 1:**
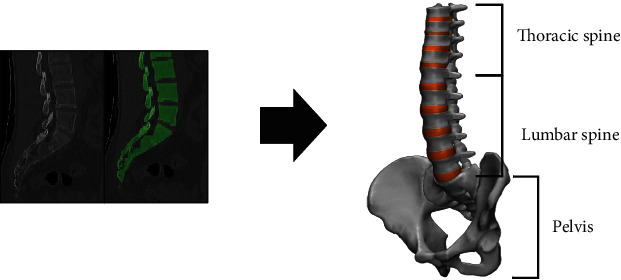
Construction of a 3-dimensional spine model using computed tomography data.

A three-dimensional (3D) thoracolumbar spine and pelvis model was constructed using computed tomography data from a normal 57-year-old male, with the slice thickness of 2-mm, along with a previously validated lumbar spine model called T9-Pelvis (Figure 1) [17]. All model moieties were created using 0.3 mm tetrahedral mesh. The mesh convergence in the present study was decided among varying element sizes. With an element size of 0.3 mm, the stress converged properly. Finally, a 0.3 mm element size was applied in our study. The finite element model consisted of the thoracolumbar and pelvic bone, intervertebral disc, and major ligaments. The number of nodes was 250,495, and the number of elements was 1,307,709 for the intact model.

We used Abaqus (version 6.14; Dassault Systems, France) to perform finite element analysis and computer-aided engineering of the spine model. We constructed the thoracolumbar spine ligaments as a truss element including the anterior longitudinal ligament, posterior longitudinal ligament, ligamentum flavum, intertransverse ligament, interspinous ligament, supraspinous ligament, and capsular ligament. We constructed the pelvic ligaments as a spring including the anterior sacroiliac ligament, posterior sacroiliac ligament, interosseous sacroiliac ligament, sacrospinous ligament, sacrotuberous ligament, superior pubic ligament, arcuate ligament, inguinal ligament, and iliolumbar ligament. The Young's modulus, Poisson's ratio, and stiffness coefficient were applied to each moiety using previously published data in the literature (Tables 1 and 2) [18–23].

We used two methods to validate the finite element analysis results. (1) The lumbar lordosis angle of the 3D model was measured and compared with the angle in the normal range [24]. (2) The range of motion (ROM) of the thoracic spine was measured by applying a pure moment (7.5 Nm) suitable for spinal mobility, and the results were compared with those in the literature [25, 26].

### 2.2. Finite Element Analysis of Surgical Models

We produced the surgical model by importing the oblique lateral interbody fusion (OLIF) and posterior lumbar interbody fusion (PLIF) cage (GS Medical, Osong, Korea) into Abaqus. The OLIF/PLIF cage dimensions were 22 mm/26 mm in length, 22 mm/14 mm in height, and 12°/6° in angle measure, respectively, according to the lumbar model height and angle. The discs of each intact lumbar segment were removed; OLIF cages were inserted into L1-L5, and PLIF cages were inserted into L5-S1. The pedicle screw system (GS Medical) was used for posterior fixation: 6.5 mm in diameter and 45 mm in length for the spine and 8.5 mm in diameter and 80 mm in length for the ilium. The pedicle screw was inserted through the center of the pedicle and parallel to the endplate, and the iliac screw was inserted through S2 ala (S2 alar-iliac screw) [27]. Next, a 6.0 mm diameter rod was constructed and attached according to the position of the pedicle screw housing. The following material properties of Grade 5 titanium (Ti-6Al-4 V) were used for the model analysis: Young's modulus = 110,000 MPa; Poisson's ratio = 0.35 [28].

**Figure 2:**
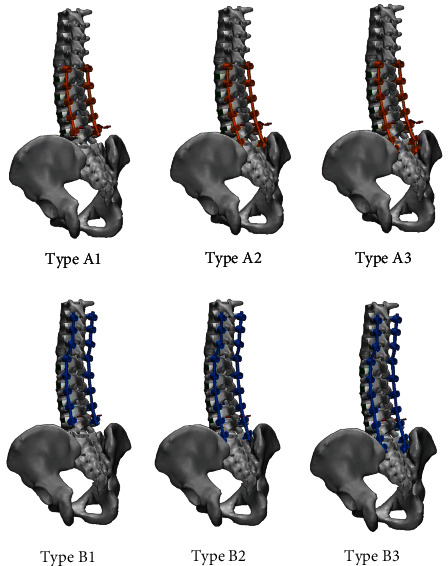
Construction of six different surgical model types for comparative analysis of the fusion level: Type A1, L1-L5 fusion; Type A2, L1-S1 fusion; Type A3, L1-ilium fusion; Type B1, T10-L5 fusion; Type B2, T10-S1 fusion; Type B3, T10-ilium fusion.

Six different fusion types for comparative analysis were made. Type A and B referred to the group of which upper fusion level was L1 and T10, respectively. Subtype 1, 2, and 3 referred to the group of which lower fusion level was L5, S1, and ilium, respectively: Type A1, L1-L5; Type A2, L1-S1; Type A3, L1-ilium; Type B1, T10-L5; Type B2, T10-S1; Type B3, T10-ilium (Figure 2).

### 2.3. Loading and Boundary Conditions

**Figure 3:**
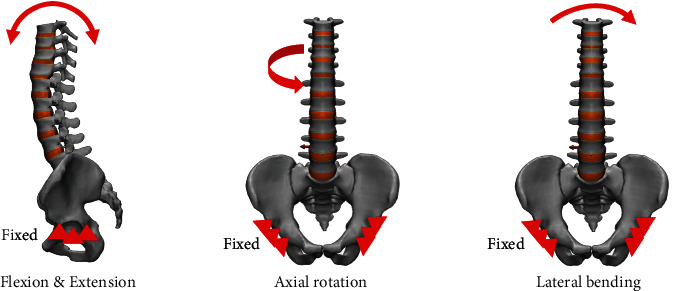
Application of a physiological load to the spine model.

To apply a physiological load, both acetabulum bones were fixed in all degrees of freedom, assuming standing on two legs. A pure moment of 10 Nm was applied to the uppermost segment (T9) endplate in the direction of flexion, extension, axial rotation, and lateral bending according to the spinal mobility. A compressive follower load of 400 N was applied according to the curvature of the vertebral segment (Figure 3) [29]. Tie contact conditions were applied assuming that the bone-implant and implant-implant interfaces were completely fused and fixed. Since the size of the finite element model was large, we tried to simplify it and show the potential risk of screw loosening using analysis of the stress occurring in the screw-bone interface.

## 3. Results

### 3.1. Peak Von Mises Stress at the Bone-Screw Interface

**Figure 4:**
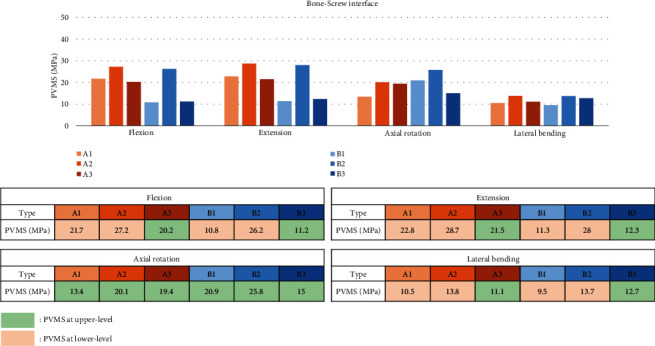
Peak von Mises stress measured at the bone-screw interface.

Stress at the bone-screw interface of type B3 was lowest in overall motions. Subtype 2 showed higher peak von Mises stress (PVMS) than subtype 1 and 3. For flexion and extension, subtype 1 and 3 showed similar results within type A and B group while type A showed higher PVMS than type B. For axial rotation, type B1 showed higher PVMS than B3. PVMS was noted at the upper end level in subtype 3 and axial rotation motion in all types, while PVMS was noted at the lower end level in other types and motions (Figure 4).

### 3.2. Peak Von Mises Stress at the Screw

**Figure 5:**
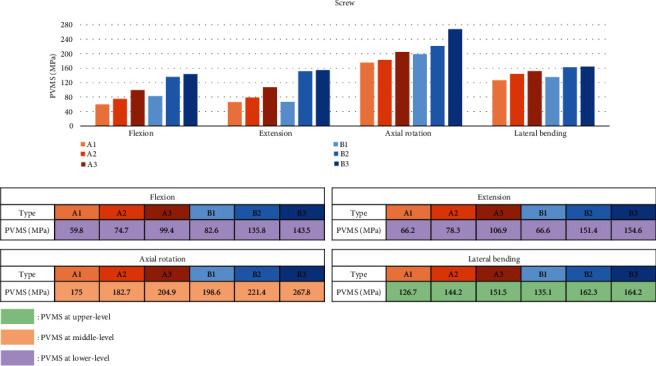
Peak von Mises stress measured at the screw.

**Figure 6:**
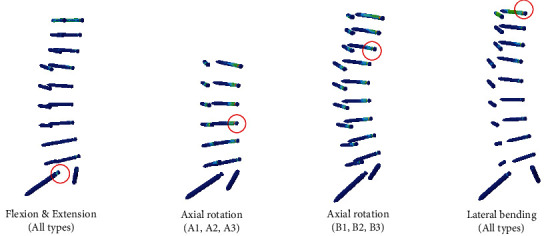
Location of peak von Mises stress at the screw.

PVMS at the screw increased as the lower fusion level changed from L5 to S1 and the ilium. Type B group showed slightly higher results in all motions than the type A group. The highest PVMS was identified in axial rotation motion, and type B3 showed the highest PVMS (267.8 MPa) among the six types. The measured stress in all motion and types was less than the yield strength (880 MPa) of Grade 5 titanium (Ti-6Al-4 V) (Figure 5). Among all the types, the highest PVMS occurred at the neck of the lowest screw in flexion and extension and at the neck of uppermost screw in lateral bending. In axial rotation, the highest PVMS occurred at the neck of L4 screw in type A group and at the neck of T12 screw in type B group (Figure 6).

### 3.3. Range of Motion at Adjacent Segments

**Figure 7:**
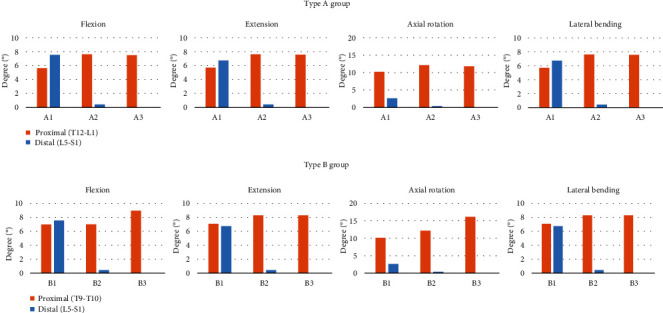
Range of motion at the proximal and distal junctions.

Overall, the proximal ROM increased as the lower fusion level changed from L5 to S1 and the ilium. For flexion, extension, and lateral bending, type B group showed similar or slightly higher proximal ROM than each corresponding type A. For axial rotation, type B3 showed higher proximal ROM (16.2°) than type A3 (11.8°). Despite interbody fusion with two cages in the L5-S1 segment, fine motion in L5-S1 was identified in subtype 2 group, whereas subtype 3 group showed no motion (Figure 7).

## 4. Discussion

Advantages of multilevel lumbar fusion with posterior pedicle screw fixation have been reported in numerous studies, and possible complications also have been researched using various methods. However, to our knowledge, there has not been a comprehensive study on biomechanical properties of different fusion levels using 3-dimensional finite element method. We found that adding iliac screws and increasing the fusion level to T10-ilium may lower the risk of screw loosening. Second, in terms of screw failure and proximal ASD, T10-ilium fusion model showed higher potential risk compared with other fusion types. Based on these results, it can be suggested that T10-ilium fusion is recommendable for spinal deformity correction and maintaining the stability, although there is a potential risk of screw failure and proximal ASD; therefore, surgeons should provide adequate patient education regarding screw failure and proximal ASD, when performing T10-ilium fusion.

In terms of screw loosening, we found that type B3 was most appropriate in overall motions. The stress at the bone-screw interface in fusion down to S1 is 58% higher on average than that in fusion down to L5 or the ilium regardless of the upper fusion level. This result was consistent with previous literature findings [30–33]. Also, type A group showed 61% higher PVMS on average than type B group in flexion and extension, possibly due to high loading at the remaining mobile T12-L1 segment. In axial rotation, type B3 showed 28% superior result compared with type B1, indicating the strong fixation effect of axial movement by iliac screws (Figure 4). The use of iliac screw has been shown to improve biomechanical stability. Yasuda et al. reported various complications including loosening, 76% in noniliac fusion group and 12% in iliac fusion group [34]. Tsuchiya et al. reported that there was no case of screw loosening with multilevel fusion using iliac screw after 5 to 10 years of follow up [35]. In this study, we revealed biomechanical superiority of the use of iliac screws using 3-dimensional finite element method. Our results would be useful when deciding proper fusion level and instrumentation method in multilevel lumbar fusion surgery.

The risk of screw failure showed increasing pattern as the upper and lower levels extended in all motions (Figure 5). PVMS was identified at the neck of the screw in all motions and types, possibly due to the smaller diameter of the screw neck part (Figure 6). This was in accordance with the study conducted by Chen et al. [36]. The measured stress in all motion and types was less than the yield strength (880 MPa) of Grade 5 titanium (Ti-6Al-4 V), implying the actual risk of screw breakage was not significantly high. Natarajan et al. indicated that the maximum von Mises stress occurs in the caudal portion of multilevel lumbar fusion [37]. In this study, we found that middle level and upper-level screws are also susceptible to failure in axial rotation and lateral bending motion, respectively.

Regarding lumbosacral junctional failure, we identified the superiority of fusion to the ilium compared with that to S1 using ROM analysis. Several previous studies have reported that extending the fusion to the ilium may prevent complications that arise from fusion to only S1 [16, 34, 35]. In this study, we found that multilevel fusion down to only S1 without iliac screws has micromotion, with an average of 0.4°, in the L5-S1 segment compared with fusion to the ilium with iliac screws (Figure 7).

The risk of proximal ASD increased as the fusion range increased to S1 and the ilium. The overall proximal ROM of type B group was similar or slightly higher, with an average of 13%, than that of type A group, a finding that was somewhat different from that of a previous clinical study [38]. Interestingly, type B3 had markedly higher (37%) proximal ROM than type A3 in axial rotation (Figure 7). A possible explanation may be that it is due to the difference in facet orientation between thoracic and lumbar vertebrae [39]. Because the facet orientation is parallel to the coronal plane, thoracic vertebrae have more rotation ability than lumbar vertebrae. When all the joints below T10 are fused, the T9-10 junction is more vulnerable in rotation motion owing to the high loading and rotation ability. Therefore, patient education regarding restricting excessive axial rotation movement would be helpful in T10-ilium fusion patients.

This study has several clinically relevant findings. Surgeons can determine the surgical outcome of patients according to various fusion levels. The risk of screw loosening and failure was numerically identified and can be applied to clinical practice. Furthermore, our results can be used as material for patient education.

Our study has limitations. First, we did not change the rod structure or material, such as the cobalt chrome rod or multiple-rod constructs. We considered the most used material and surgical technique to date to limit the variables. Further studies are needed to confirm the effect of the rod structure. Second, our model is based on a normal male individual; thus, it does not reflect the status of undercorrection or overcorrection and the poor bone quality observed in patients. Constructing a spine model based on a real patient with adult spinal deformity remains technically challenging. Further technical research is required to construct a real deformed spine model. In addition, the models in this study were considered as linear since we intended to see the trends between the models. We will apply the anisotropic properties of the bone in the future research. Finally, we assumed the screw-bone interface to be completely fused and fixed state and simplified the screw threads as previous finite element studies [40–45] since the size of the finite element model was large, and the screw loosening was thought to be a result of accumulated damage in the screw-bone interface for a long-term period. A more complex finite element model of a surface-to-surface contact with a coefficient friction between the screw-bone interface will be applied in the future study.

## 5. Conclusions

In multilevel lumbar fusion surgery for adult spinal deformity, adding iliac screws and increasing the fusion level to T10-ilium may lower the risk of screw loosening. In terms of screw failure and proximal ASD, however, T10-ilium fusion has a higher potential risk compared with other fusion types. These results will contribute for surgeons to provide adequate patient education regarding screw failure and proximal ASD, when performing multilevel lumbar fusion.

## Figures and Tables

**Table 1:** Material properties of the spine.

Materials	Young's modulus (Mpa)	Poisson's ratio
Ilium (cortical)	17,000	0.3
Ilium (cancellous)	132	0.2
Sacrum (cortical)	6140	0.3
Sacrum (cancellous)	1400	0.3
Vertebral body (cortical)	12,000	0.3
Vertebral body (cancellous)	100	0.3
Posterior elements	3500	0.25
Annulus fiber	450	
Annulus matrix	4.2	0.45
Nucleus pulposus	1	0.499
Endplate	100	0.3

**Table 2:** Material properties of the pelvic ligaments.

Materials	Stiffness coefficient (*N*/mm)
Anterior sacroiliac ligament	700
Posterior sacroiliac ligament (long)	1000
Posterior sacroiliac ligament (short)	400
Interosseous sacroiliac ligament	2800
Sacrospinous ligament	1400
Sacrotuberous ligament	1500
Superior pubic ligament	500
Arcuate pubic ligament	500
Inguinal ligament	250
Iliolumbar ligament	1000

## Data Availability

The datasets generated and/or analyzed during the current study are not publicly available due to the collaborative achievement related patent issue with collaborating research institute but are available from the corresponding author on reasonable request.
